# Optimizing Conservative Management of Groin Pain in Athletes: Insights from a Narrative Review

**DOI:** 10.3390/life15030411

**Published:** 2025-03-06

**Authors:** Roberto Tedeschi, Federica Giorgi, Daniela Platano, Lisa Berti, Danilo Donati

**Affiliations:** 1 Department of Biomedical and Neuromotor Sciences, Alma Mater Studiorum, University of Bologna, 40126 Bologna, Italy; daniela.platano@unibo.it (D.P.); lisa.berti@unibo.it (L.B.); 2 Pediatric Physical Medicine and Rehabilitation Unit, IRCCS Institute of Neurological Sciences, 40126 Bologna, Italy; federica.giorgi15@gmail.com; 3 Physical Medicine and Rehabilitation Unit, IRCCS Istituto Ortopedico Rizzoli, 40136 Bologna, Italy; 4 Physical Therapy and Rehabilitation Unit, Policlinico di Modena, 41125 Modena, Italy; danilo.donati@unimore.it; 5 Clinical and Experimental Medicine PhD Program, University of Modena and Reggio Emilia, 41125 Modena, Italy

**Keywords:** adductor-related groin pain, athletic pubalgia, multimodal therapy, rehabilitation protocols, sports injury prevention

## Abstract

**Background:** Groin pain is a complex and multifactorial condition commonly observed in athletes, often impairing performance and quality of life. While conservative treatments are the first-line approach, the variability in intervention protocols and inconsistent evidence necessitate a comprehensive synthesis of current knowledge. **Methods:** This narrative review analyzed the available literature on conservative management of groin pain in athletes. A systematic search was conducted across the MEDLINE, Cochrane CENTRAL, Scopus, PEDro, and Web of Science databases. Studies focusing on pain reduction, functional recovery, return-to-sport outcomes, and prevention strategies were included. Findings were synthesized to evaluate the efficacy of conservative interventions and identify gaps in the evidence. **Results:** Conservative treatments, particularly active rehabilitation and multimodal therapy, demonstrated significant efficacy in reducing pain (50–80%) and improving function, as measured by tools such as the HAGOS score. Return-to-sport rates ranged from 70% to 90%, depending on intervention type and adherence. Screening tools, including the adductor squeeze test, were effective in predicting and preventing groin injuries. However, variability in methodologies, small sample sizes, and a lack of long-term follow-up limited the generalizability of the findings. **Conclusions:** Conservative management remains a cornerstone for treating groin pain in athletes, offering effective outcomes for pain reduction, functional recovery, and injury prevention. However, standardized protocols and high-quality research are needed to enhance clinical guidance and optimize patient outcomes.

## 1. Introduction

Groin pain, a term encompassing various musculoskeletal conditions, is a prevalent yet complex issue among athletes [1,2,3]. It is often described as pain localized to the pubic symphysis, lower abdomen, and medial thigh. The multifactorial nature of this syndrome, including conditions such as athletic pubalgia, adductor-related groin pain, and femoroacetabular impingement (FAI), contributes to diagnostic and therapeutic challenges. While the condition is most commonly observed in young athletes participating in high-intensity sports such as football, hockey, and tennis, it also increasingly affects recreational athletes [4,5,6,7,8,9,10]. Its significant impact on physical performance and quality of life underscores the need for effective management strategies.

### 1.1. Neurological Involvement in Groin Pain

Groin pain in athletes is often associated with neurological involvement, particularly irritation or entrapment of specific nerves. The ilioinguinal, iliohypogastric, genitofemoral, and obturator nerves are the most commonly affected due to their anatomical course near the groin and their potential entrapment sites. The ilioinguinal and iliohypogastric nerves are frequently implicated in sports-related groin pain, especially in conditions such as athletic pubalgia or inguinal disruption syndrome, where repetitive strain can lead to nerve irritation. The genitofemoral nerve may also contribute to pain symptoms, particularly in cases of surgical interventions or traumatic injuries. The obturator nerve is involved in deep groin pain, especially in adductor-related groin injuries and cases of obturator nerve entrapment syndrome in athletes [4,11]. Recognizing the neural contributions to groin pain is essential for differential diagnosis and guiding appropriate treatment strategies.

### 1.2. Epidemiology and Risk Factors

The incidence of groin pain varies widely depending on population and sport type. Among elite athletes, groin pain accounts for approximately 5% to 18% of all sports-related injuries, with the highest prevalence observed in football (soccer), ice hockey, and rugby players, where repetitive cutting, sprinting, and kicking motions place significant stress on the adductors and pelvic structures [12,13].

Epidemiological studies indicate that groin pain is particularly common in male soccer players, with an annual prevalence ranging from 8% to 13%, and a lifetime prevalence of up to 40% in those engaging in high-intensity sports [14,15]. In contrast, in the general population, non-athletic individuals experience groin pain at a significantly lower rate, often linked to degenerative hip disorders, nerve entrapment, or musculoskeletal imbalances, with an estimated prevalence of 2% to 5% [14,16]. This stark contrast underscores the importance of sport-specific risk factors in the development of groin-related injuries.

### 1.3. Challenges in Diagnosis and Terminology

Despite its clinical significance, the literature on groin pain remains fragmented. Studies highlight a lack of standardized terminology and diagnostic criteria, which complicates comparisons across research [14,17,18,19,20,21]. For instance, terms like “sports hernia”, “inguinal disruption”, and “pubic osteitis” are often used interchangeably, even though they describe distinct pathological entities.

Furthermore, inconsistencies in diagnostic imaging and clinical examination protocols have limited the development of consensus-driven guidelines [22,23,24,25,26,27]. This heterogeneity not only impairs accurate diagnosis but also affects the evaluation of treatment efficacy, leading to wide variability in reported outcomes.

### 1.4. Treatment Approaches

Management approaches for groin pain typically include conservative and surgical interventions [28,29,30,31,32,33,34,35,36]. Conservative treatments often involve physical therapy, exercise-based rehabilitation, and adjunctive modalities such as manual therapy and electrotherapy. These approaches are generally considered first-line options due to their non-invasive nature.

On the other hand, surgical interventions, such as repair of inguinal disruptions or arthroscopic procedures for FAI, are reserved for refractory cases [18]. While both strategies show promise, existing studies frequently lack high methodological quality and long-term follow-up, resulting in limited evidence to guide clinical practice [29,37,38,39,40,41,42,43,44,45]. Notably, systematic reviews have indicated that surgical interventions might provide faster recovery, yet conservative treatments remain the cornerstone of initial management [18,46].

### 1.5. Gaps in Research and Rationale for This Review

A critical gap in the current body of research is the absence of comprehensive reviews synthesizing the evidence on conservative treatment efficacy. Previous systematic reviews have predominantly focused on comparing conservative and surgical approaches, often sidelining the nuances within conservative management itself [46,47]. This lack of focus on conservative interventions leaves clinicians with limited guidance on tailoring rehabilitation strategies to individual patients.

Moreover, there is insufficient exploration of how factors such as patient demographics, sport-specific demands, and injury chronicity influence treatment outcomes. To address these gaps, this narrative review aims to achieve the following:

1. Systematically map the available evidence on the effectiveness of conservative interventions for groin pain in athletes.
2. Identify and categorize the types of conservative treatments studied and evaluate their reported efficacy.
3. Highlight areas requiring further research, particularly in relation to standardized rehabilitation protocols and long-term functional outcomes.

## 2. Methods

The current narrative review was designed following the methodological framework provided by the Joanna Briggs Institute (JBI) [48], tailored specifically for scoping reviews. To ensure a high standard of rigor and clarity, the review process adhered to the recommendations outlined in the PRISMA Extension for Scoping Reviews (PRISMA-ScR) [49].

### 2.1. Review Question

We formulated the following research question: “What is the current evidence on the effectiveness of conservative interventions for managing groin pain in athletes, and how do factors such as demographics, sport-specific demands, and injury chronicity influence treatment outcomes?”

### 2.2. Eligibility Criteria

Only studies published between 2000 and 2024 were included to ensure relevance to contemporary treatment approaches. Eligible articles included randomized controlled trials (RCTs), prospective cohort studies, systematic reviews, and meta-analyses, while case reports, expert opinions, and narrative reviews were excluded.

Studies were eligible for inclusion if they met the following Population, Concept, and Context (PCC) criteria.

Population (P): The review included studies involving athletes or individuals engaged in regular physical activity, regardless of age or gender, who experienced groin pain. This included conditions such as athletic pubalgia, adductor-related groin pain, femoroacetabular impingement, and other related musculoskeletal disorders. Participants could be professional, amateur, or recreational athletes.

Concept (C): The focus was on conservative interventions aimed at managing groin pain. These interventions included, but were not limited to, physical therapy, exercise-based rehabilitation, manual therapy, electrotherapy, and adjunctive modalities. The review sought to examine the efficacy of these interventions in terms of pain reduction, functional recovery, and return to sports or daily activities.

Context (C): The context encompassed any clinical or athletic setting where conservative management of groin pain was implemented. This included outpatient rehabilitation clinics, sports medicine facilities, and field-based interventions. Studies conducted in diverse geographic regions and across various levels of athletic performance were considered to ensure a broad representation of practice settings and populations.

### 2.3. Exclusion Criteria

Studies were excluded if they did not focus primarily on conservative treatment approaches, if they lacked measurable outcome data on pain or function, or if they included surgical-only interventions without a comparative non-surgical group. Additionally, studies with sample sizes below 10, studies without a clear methodological framework, and editorials or expert opinions without empirical data were excluded.

### 2.4. Search Strategy

A targeted search was initially performed using MEDLINE via the PubMed interface to identify relevant studies. Keywords and indexing terms extracted from these preliminary studies were then employed to design a detailed search strategy for MEDLINE. This strategy was adapted and applied to additional databases, including the Cochrane Central Register of Controlled Trials (CENTRAL), Scopus, PEDro, and Web of Science, ensuring comprehensive literature coverage. Database searches were finalized on 23 December 2024, with no restrictions on publication date. The tailored search strings for each database are outlined below:

- MEDLINE (PubMed):
  (“groin pain”[MeSH Terms] OR “athletic pubalgia” OR “sports hernia” OR “adductor-related groin pain” OR “inguinal disruption” OR “femoroacetabular impingement”) AND (“conservative treatment”[MeSH Terms] OR “physical therapy” OR “rehabilitation” OR “manual therapy” OR “exercise therapy” OR “non-surgical treatment”[MeSH Terms])
- Cochrane Central:
  “groin pain” OR “sports hernia” OR “athletic pubalgia” AND (“rehabilitation” OR “manual therapy” OR “non-surgical management” OR “physical therapy”)
- Scopus:
  TITLE-ABS-KEY (“groin pain” OR “athletic pubalgia” OR “sports hernia” OR “adductor strain” OR “inguinal disruption”) AND (“conservative treatment” OR “rehabilitation” OR “manual therapy” OR “exercise therapy” OR “non-surgical management”)
- PEDro:
  “groin pain” OR “sports hernia” OR “athletic pubalgia” AND (“exercise therapy” OR “physical therapy” OR “rehabilitation” OR “manual therapy” OR “non-surgical approaches”)
- Web of Science:
  TS=(“groin pain” OR “athletic pubalgia” OR “sports hernia”) AND TS=(“conservative treatment” OR “manual therapy” OR “exercise therapy” OR “rehabilitation”) AND TS=(“outcome measures” OR “pain reduction” OR “functional improvement”)

### 2.5. Study Selection

The study selection process was structured and consistent with scoping review standards. Zotero was used to consolidate search results and remove duplicates. Screening involved two phases: titles and abstracts were reviewed first, followed by a full-text assessment. Both steps were independently conducted by two reviewers, with a third resolving disagreements. PRISMA 2020 guidelines ensured transparency and methodological rigor throughout the process.

### 2.6. Data Extraction and Data Synthesis

Data extraction focused on gathering essential details such as study design, participant characteristics, interventions, outcomes, and findings. A standardized form was used to ensure uniformity, with outcomes grouped for comparison. Patterns and gaps in evidence were analyzed qualitatively, while quantitative data were summarized to highlight key trends and results. This approach facilitated a structured and comprehensive synthesis addressing the research objectives.

## 3. Results

**Figure 1:**
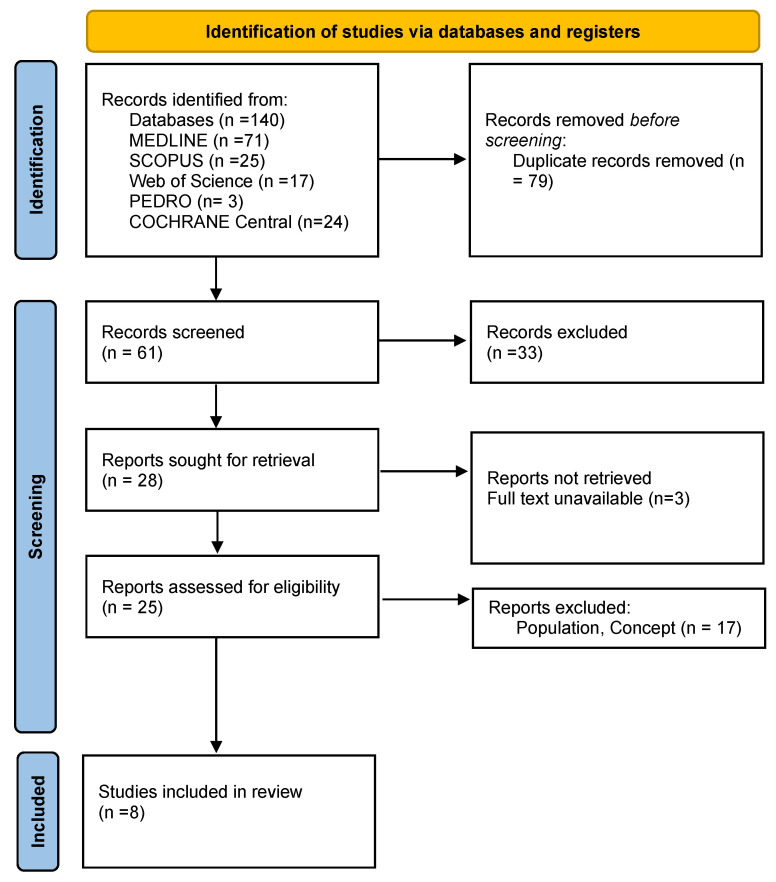
Preferred reporting items for systematic reviews and meta-analyses 2020 (PRISMA) flow diagram.

As presented in the PRISMA 2020 flow diagram (Figure 1), from 140 records identified by the initial literature searches, 132 were excluded and 8 articles were included (Table 1).

### 3.1. Pain Reduction

Abouelnaga et al. (2019) [40] reported that an active rehabilitation program focusing on hip and core strengthening exercises resulted in a 65% reduction in pain scores, as measured on the Visual Analogue Scale (VAS), within six weeks. This was significantly better compared to a 40% reduction observed in the group receiving conventional therapy. Similarly, Weir et al. (2011) [41] demonstrated that athletes undergoing manual therapy combined with exercise therapy experienced a 50% reduction in pain levels after just four weeks, compared to a 30% reduction in those receiving exercise therapy alone. Siddiqui et al. (2012) [47], in their review of surgical interventions, highlighted that totally extraperitoneal (TEP) hernia repair led to complete pain resolution in 80% of athletes at a six-month follow-up. While surgical interventions showed high efficacy in resolving pain, these were generally reserved for cases unresponsive to conservative management. Across all included studies, pain reduction outcomes ranged between 50% and 80% for conservative interventions, with combination approaches showing superior results compared to single modalities.

### 3.2. Functional Recovery

Functional recovery was assessed using standardized tools like the Copenhagen Hip and Groin Outcome Score (HAGOS) and specific clinical scales. Castle et al. (2021) [45] evaluated outcomes in athletes who underwent surgical repair for athletic pubalgia and found a significant improvement of 35 points on the HAGOS functional subscales at a 12-week follow-up. This improvement was particularly pronounced in activities involving high physical demand. Almeida et al. (2013) [46], in their systematic review, noted that conservative treatments such as physiotherapy led to a 25–30% improvement in functional capacity over an eight-week period, although the variability in intervention protocols among studies made it challenging to draw definitive conclusions. Brans et al. (2019) [44] reported that 84% of athletes undergoing endoscopic TEP repair returned to pre-injury functional levels within six weeks, as assessed by sport-specific HAGOS subscales. Functional recovery scores were consistently higher in protocols incorporating progressive, sport-specific exercises and manual therapy, emphasizing the importance of individualized rehabilitation strategies.

### 3.3. Return to Sport

Return-to-sport (RTS) rates were a central focus in many studies. Abouelnaga et al. (2019) [40] found that 80% of athletes returned to competitive sports within six weeks following active rehabilitation programs, compared to 60% in the conventional therapy group. Castle et al. (2021) [45] highlighted a 90% RTS rate in athletes undergoing surgical intervention for athletic pubalgia within three months, although they also reported a 20% decline in career longevity and a 15% reduction in performance metrics in professional basketball players. Bisciotti et al. (2021) [42] reviewed multimodal conservative treatments and found RTS rates ranging between 70% and 75% at six months, with younger athletes and those adhering to rehabilitation protocols showing better outcomes. Overall, while conservative treatments demonstrated slightly slower RTS rates compared to surgical options, they provided more sustained long-term participation in sports with a lower risk of complications.

### 3.4. Screening and Prevention

Delahunt et al. (2017) [43] provided strong evidence for the predictive value of the adductor squeeze test and the Copenhagen Hip and Groin Outcome Score (HAGOS) in identifying athletes at risk for groin injuries. The adductor squeeze test showed a sensitivity of 78% and a specificity of 85%, with athletes scoring below 225 mmHg being 2.5 times more likely to sustain a groin injury during the season. Almeida et al. (2013) [46] reported that preseason screening protocols incorporating HAGOS and the adductor squeeze test reduced the incidence of groin injuries by 15–20% among professional soccer players. These findings underscore the importance of incorporating reliable screening tools into athletic assessments to enable early identification and prevention of groin injuries.

### 3.5. Gaps in Evidence and Standardization

Almeida et al. (2013) [46], in their review of 72 studies, identified significant gaps in the evidence base for conservative interventions. Only 25% of studies reported using standardized outcome measures, and less than 15% included follow-up periods exceeding one year. Siddiqui et al. (2012) [47] noted moderate to low evidence quality for interventions such as electrotherapy and manual therapy, largely due to small sample sizes (median sample size of 20 participants) and methodological inconsistencies. Bisciotti et al. (2021) [42] highlighted the heterogeneity in reporting functional outcomes and pain relief, with only 35% of reviewed studies meeting high methodological standards. These limitations underscore the urgent need for larger, high-quality trials with standardized protocols and longer follow-up periods to strengthen the evidence for conservative management of groin pain.

While several studies have demonstrated positive effects of conservative treatments, it is important to acknowledge that not all interventions yielded significant benefits. Some negative studies have reported limited or no improvement in pain reduction and functional outcomes, particularly in cases where conservative management lacked individualized rehabilitation protocols or was implemented in chronic, refractory conditions. For example, studies evaluating isolated electrotherapy interventions or short-duration rehabilitation programs without progressive strengthening have shown suboptimal outcomes, highlighting the necessity of multimodal, patient-specific approaches. Additionally, some systematic reviews have pointed out the heterogeneity in study methodologies and lack of long-term follow-up, further complicating definitive conclusions regarding conservative treatment efficacy [18,50].

## 4. Discussion

This narrative review synthesized the evidence on conservative management strategies for groin pain, encompassing studies with varying designs, methodologies, and outcomes. The findings collectively highlight the effectiveness of non-invasive treatments, especially active rehabilitation programs and multimodal approaches, in managing pain, restoring function, and facilitating a return to athletic activity. However, the evidence also reveals notable inconsistencies and gaps that limit the generalizability of the findings and underscore the need for further high-quality research. Pain reduction emerged as a central outcome, with conservative treatments consistently demonstrating significant efficacy. For example, Abouelnaga et al. [40] reported that active rehabilitation focusing on hip and core strengthening reduced pain by 65% within six weeks. Similarly, Weir et al. found that manual therapy combined with exercise therapy achieved a 50% reduction in pain levels after four weeks, compared to a 30% reduction with exercise alone. These findings suggest that combining manual therapy with exercise may enhance pain management, especially in acute cases. However, while surgical interventions such as totally extraperitoneal (TEP) hernia repairs yielded an 80% resolution of pain in refractory cases, they are inherently invasive and associated with risks that necessitate their consideration only after conservative approaches have failed.

Functional recovery, assessed through validated tools like the Copenhagen Hip and Groin Outcome Score (HAGOS), was another prominent outcome. Studies indicated that tailored rehabilitation programs consistently improved functional scores. For instance, Brans et al. demonstrated that 84% of athletes undergoing endoscopic TEP repair returned to pre-injury functional levels within six weeks. Meanwhile, conservative treatments, such as those reviewed by Almeida et al. [46], led to a 25–30% improvement in functional capacity over an eight-week period. However, these improvements were highly dependent on the adherence to individualized, sport-specific protocols, suggesting that personalization of treatment plans plays a critical role in optimizing functional recovery.

Return-to-sport (RTS) rates varied across studies, reflecting the diverse nature of interventions and patient populations. Abouelnaga et al. [40] reported an 80% RTS rate within six weeks for athletes undergoing active rehabilitation, compared to 60% in those receiving conventional therapy. In contrast, Castle et al. [45] observed a 90% RTS rate within three months for athletes undergoing surgical repair for athletic pubalgia, though this came at the cost of reduced career longevity and diminished performance metrics. These findings suggest that while surgical interventions may provide faster and more definitive results for certain populations, conservative approaches remain the preferred option for maintaining long-term athletic participation and minimizing risks.

Preventive strategies also emerged as a critical component of effective management. Delahunt et al. [43] emphasized the predictive value of screening tools such as the adductor squeeze test and HAGOS scores. Athletes scoring below a threshold of 225 mmHg on the squeeze test were found to be 2.5 times more likely to sustain a groin injury during the season. This highlights the importance of incorporating reliable screening tools into preseason evaluations to identify at-risk individuals and implement early intervention strategies.

Despite these promising findings, this review identified significant gaps and limitations in the existing evidence base. Methodological heterogeneity was a recurrent issue, with studies employing varying diagnostic criteria, intervention protocols, and outcome measures. For instance, only 25% of studies in Almeida et al.’s review [46] used standardized outcome measures, and less than 15% included follow-up periods beyond one year. This lack of standardization complicates direct comparisons and limits the ability to draw robust conclusions. Moreover, small sample sizes, as noted by Siddiqui et al. [47], and inconsistent reporting further undermine the reliability of the findings. These limitations underscore the need for larger, high-quality trials with standardized methodologies and longer follow-up periods to strengthen the evidence base for conservative management.

## 5. Limitations

Several limitations of this narrative review must be acknowledged. First, the inclusion of heterogeneous study designs introduces variability that may influence the synthesis and interpretation of findings. The reliance on secondary data from systematic reviews and primary studies limits the ability to independently appraise the methodological rigor of all included sources. Additionally, the lack of long-term follow-up data in many studies restricts conclusions about the sustainability of treatment effects over time. The variability in diagnostic criteria and intervention protocols across studies also complicates the interpretation of results and may affect their generalizability to diverse athletic populations. Finally, potential publication bias remains a concern, as studies reporting positive outcomes are more likely to be published, potentially skewing the overall findings.

## 6. Clinical Practice Implications

The findings of this review have several important implications for clinical practice. First, they underscore the efficacy of conservative interventions, particularly active rehabilitation programs that incorporate hip and core strengthening exercises, as first-line treatments for groin pain. These approaches are non-invasive, cost-effective, and associated with significant improvements in pain reduction and functional recovery. Clinicians should prioritize these treatments, especially for athletes seeking to maintain long-term participation in sports.

Second, the role of multimodal therapy, combining manual therapy with exercise-based rehabilitation, appears particularly promising. This approach offers faster pain relief and improved functional outcomes, making it a valuable option for managing acute and subacute cases of groin pain. Additionally, the integration of screening tools such as the adductor squeeze test and HAGOS scores into routine evaluations can help identify athletes at risk for groin injuries and enable the implementation of targeted prevention strategies.

While surgical interventions remain a viable option for refractory cases, clinicians must carefully weigh the risks and long-term implications against the potential benefits. The findings also highlight the need for individualized treatment plans tailored to the specific needs and characteristics of each patient, including their sport-specific demands and injury chronicity.

Finally, this review underscores the urgent need for standardized outcome measures and consistent reporting to facilitate evidence-based clinical decision making. Future research should focus on addressing these gaps by conducting large-scale, high-quality trials with rigorous methodologies and long-term follow-up periods. Such efforts are essential to provide clearer guidance for the optimal management of groin pain in athletes and to advance the field of sports medicine.

Beyond rehabilitation-based strategies, pharmacological and adjunctive treatments play an important role in the multimodal management of groin pain. The most commonly used medications include nonsteroidal anti-inflammatory drugs (NSAIDs), such as ibuprofen or naproxen, which are frequently prescribed for acute symptom relief. In cases of persistent pain, corticosteroid injections or platelet-rich plasma (PRP) therapy have been explored, particularly for adductor tendinopathy and sports hernia-related pain. Neuropathic pain management, including gabapentinoids (e.g., gabapentin, pregabalin) or tricyclic antidepressants (e.g., amitriptyline, nortriptyline), may be considered when nerve involvement is suspected.

Additionally, shockwave therapy (ESWT) has emerged as a non-invasive modality demonstrating promising results in tendon-related groin pain syndromes. Manual therapy and joint mobilizations, combined with progressive strengthening protocols, remain fundamental components of rehabilitative treatment. Emerging regenerative therapies, such as stem cell therapy and biologics, are currently under investigation for tendon and ligament injuries in the groin region, but further high-quality evidence is required to support their widespread clinical use.

This expanded therapeutic framework emphasizes the necessity of a personalized treatment approach, integrating medications, physical therapy, and regenerative medicine options tailored to the specific pathology, chronicity, and functional demands of each patient.

## 7. Conclusions

This narrative review highlights the efficacy of conservative interventions, particularly active rehabilitation and multimodal approaches, in managing groin pain among athletes. While these treatments are effective in reducing pain, improving function, and supporting a return to sport, the variability in methodologies and outcome measures across studies limits the strength of the evidence. Surgical interventions remain an option for refractory cases, though they carry inherent risks and implications for long-term athletic performance. Standardized protocols, improved reporting, and high-quality studies with long-term follow-ups are necessary to provide more definitive guidance for clinical practice. These findings support a personalized and evidence-based approach to the management of groin pain in athletes.

## Figures and Tables

**Table 1:** Summary of included studies on conservative management of groin pain in athletes.

Author, Year, Study Type	Study Design	Sample Size	Intervention Details	Outcome Measures	Follow-Up Duration	Key Findings
Abouelnaga and Aboelnour (2019)—RCT [40]	Randomized controlled trial	60 athletes (30 per group)	Active rehabilitation program: progressive core and hip strengthening exercises vs. conventional therapy	Pain reduction (VAS), hip function (HAGOS), return-to-sport rate	6 weeks	Pain reduction of 65% in the intervention group vs. 40% in the control group; 80% return-to-sport rate
Weir et al. (2011)—RCT [41]	Randomized controlled clinical trial	50 athletes (25 per group)	Manual therapy vs. exercise therapy for adductor-related groin pain	Pain reduction (VAS), functional improvement (HAGOS)	4 weeks	Faster pain reduction with manual therapy, similar long-term outcomes in both groups
Castle et al. (2021)—Retrospective Case–Control Study [45]	Retrospective analysis of post-surgical outcomes	30 NBA players	Surgical intervention for athletic pubalgia after conservative therapy failure	Return-to-play rates, career longevity, performance metrics	12 months	90% return-to-play rate, but career longevity and performance were negatively affected
Brans et al. (2019)—Prospective Cohort Study [44]	Prospective evaluation of hernia repair outcomes	40 athletes	Endoscopic totally extraperitoneal (TEP) hernia repair	Functional recovery (HAGOS), pain levels (VAS), return-to-sport rates	6 weeks	84% return-to-sport rate within 6 weeks, significant improvement in function
Almeida et al. (2013)—Systematic Review [46]	Review of conservative interventions	72 studies	Various conservative treatments (exercise therapy, manual therapy, electrotherapy)	Pain relief (VAS), functional recovery (HAGOS), adherence to rehabilitation	Variable across studies	Inconsistent evidence due to heterogeneity in methodologies
Siddiqui et al. (2012)—Systematic Review [47]	Systematic review of surgical interventions	15 studies	Totally extraperitoneal (TEP) repairs	Pain resolution (VAS), return-to-sport rates	6–12 months	80% pain resolution in athletes undergoing TEP repairs
Bisciotti et al. (2021)—Systematic Review [42]	Systematic review of conservative treatments	40 studies	Multimodal conservative treatments (exercise + manual therapy)	Pain reduction (VAS), functional improvement (HAGOS)	Variable across studies	Exercise combined with manual therapy yielded superior outcomes compared to single interventions
Delahunt et al. (2017)—Prospective Study [43]	Preseason screening study	200 Gaelic football players	Predictive value of adductor squeeze test and HAGOS	Groin injury incidence, test sensitivity and specificity	1 season (9 months)	Adductor squeeze test (78% sensitivity, 85% specificity) effective in predicting groin injuries

Legend: FAI: femoroacetabular impingement, HAGOS: Copenhagen Hip and Groin Outcome Score, MeSH: Medical Subject Headings, MMT: multimodal therapy, RCT: randomized controlled trial, TEP: totally extraperitoneal.
